# Integrative Genome-Wide Association and Transcriptome Analyses Identify Candidate Genes for Salt Tolerance During Cotton Germination

**DOI:** 10.3390/plants15060937

**Published:** 2026-03-19

**Authors:** Yin Wang, Yilei Long, Shen Jin, Yinan Yang, Shixiao Fang, Xiutong Wu, Teng Liu, Xiantao Ai

**Affiliations:** 1 College of Life Sciences and Technology, Xinjiang University, Urumqi 830017, China; 107552301030@stu.xju.edu.cn (Y.W.); longyilei2021@163.com (Y.L.); 107552400997@stu.xju.edu.cn (S.J.); 107552401014@stu.xju.edu.cn (Y.Y.); 2 College of Agriculture, Xinjiang Agricultural University, Urumqi 830052, China; 18158802309@163.com (S.F.); crpc1122@163.com (X.W.); liuliuqiu1130@163.com (T.L.); 3 College of Smart Agriculture (Research Institute), Xinjiang University, Urumqi 830017, China

**Keywords:** *Gossypium hirsutum*, salt tolerance, GWAS, transcriptomics, candidate genes, seed germination

## Abstract

Genome-wide association analysis and transcriptomics were used to investigate salt tolerance traits during germination in 300 *Gossypium hirsutum* L. germplasm accessions, with the objective of identifying genes and molecular markers associated with salt tolerance. Under 200 mmol L^−1^ NaCl stress, six traits were evaluated, germination rate, root length, shoot length, root fresh weight, shoot fresh weight, and total fresh weight, as well as their respective salt tolerance indices. A total of 1277 significantly associated single-nucleotide polymorphism (SNP) markers were identified and mapped to 94 quantitative trait loci (QTLs). Of these, 49 QTLs were detected by three or more analytical models, and three QTLs were prioritized for further investigation. Subsequent analysis of these QTLs identified 73 candidate genes potentially involved in cotton salt tolerance. Integration of transcriptomic data revealed that three candidate genes were among the differentially expressed genes (DEGs). Examination of their RNA-seq expression profiles demonstrated significant differences in fragments per kilobase of transcript per million mapped reads (FPKM) values across sampling time points. These three candidate genes are therefore predicted to be associated with salt tolerance during cotton germination. The results provide new insights into the molecular regulatory mechanisms of salt stress tolerance in cotton and offer valuable genetic resources and molecular markers for the genetic improvement of salt tolerance.

## 1. Introduction

Soil salinization represents a significant barrier to agricultural development. The intensification of this process, driven by global climate change and human activities, has accelerated the depletion of arable land resources [1]. Crucially, seed germination represents the most critical bottleneck for successful crop establishment, as it marks the sensitive transition from a quiescent state to active growth. In saline and drought-prone environments, the adaptability of seeds during this early phase is the primary determinant of plant survival [2,3]. Saline–alkali stress severely impairs plant growth, development, and cellular metabolism [4] by disrupting ionic permeability and osmotic balance. For cotton (Gossypium), which is frequently cultivated in regions characterized by high soil salinity, resistance during the germination and seedling stages is particularly vital yet constitutes the weakest link in its life cycle [5,6]. High concentrations of soluble salts decrease soil osmotic potential, significantly impairing water uptake by seeds and roots [7]. This disruption of ion homeostasis interferes with essential metabolic processes and substantially reduces root vitality and photosynthetic capacity [8]. While cotton’s salt tolerance typically increases after the three-leaf stage [9], the damage sustained during germination directly influences the population density, subsequent vegetative growth, and ultimate fiber quality [10]. Therefore, identifying cotton germplasm with robust salt tolerance during the germination stage is a prerequisite for enhancing crop resilience and securing yields in marginal lands.

Salt stress is among the most detrimental abiotic factors affecting plant productivity. Globally, saline–alkali soils cover approximately 7% of terrestrial land, and in China, the area of saline–alkali land exceeds 100 million hectares and continues to expand [11]. Under high-salt stress, plants have evolved complex regulatory networks to enhance survival [12]. Plants growing in saline–alkali soils experience severe ionic stress, which disrupts normal ion distribution and undermines water potential homeostasis [13]. Cotton (Gossypium) is the world’s most significant natural fiber crop and a cornerstone of the global textile industry, possessing immense strategic and economic value. As a moderately salt-tolerant species, cotton plays a vital role in utilizing marginal lands and maintaining ecological stability in arid regions [14]. In China, cotton cultivation remains a national priority; according to the National Bureau of Statistics, the planting area in 2025 reached 2979.2 thousand hectares, with a total output of 6.641 million tons—a 7.7% increase over 2024. Notably, the Xinjiang region now accounts for 90.2% of China’s total cotton production [15]. underscoring the crop’s concentration in areas characterized by saline–alkali soils. While the deployment of insect-resistant and nitrogen-efficient varieties has enhanced the crop’s environmental sustainability [16], excessive salt stress remains a primary constraint. Recent studies establish a salinity threshold of 7.7 dS m^−1^ for cotton [17,18]; beyond this point, yields decline sharply, with a 0.5% soil salt content potentially leading to a 50% reduction in productivity [19]. Consequently, deciphering the mechanisms of salt tolerance during the sensitive germination stage is essential for securing global cotton supplies and optimizing land-use efficiency.

Genome-wide association studies (GWASs), which rely on linkage disequilibrium (LD), employ high-density molecular markers to identify loci associated with phenotypic variation, thereby elucidating the genetic basis of complex traits [5]. Since Risch first introduced the concept [20], GWASs have demonstrated reliability in identifying candidate genes across various species [21,22]. In recent years, GWASs have been extensively applied to crops such as rice [23], soybean [24], and maize [25]. The refinement of cotton genetic maps and advancements in statistical analysis software have facilitated the broader application of GWAS in cotton research [26]. Salt tolerance in cotton is a polygenic quantitative trait, and candidate genes can be effectively identified through quantitative trait locus (QTL) mapping. For instance, Yuan et al. [27] conducted GWAS on 196 upland cotton accessions to identify QTLs and candidate genes associated with salt tolerance during germination, reporting 33 significant single-nucleotide polymorphisms (SNPs), including 17 detected in at least two environments. While these foundational studies provide valuable insights, numerous GWASs have predominantly focused on terminal traits such as yield and fiber quality. Consequently, the genetic architecture of salt tolerance specifically during the cotton germination stage remains insufficiently characterized. Furthermore, previous efforts have largely relied on single-omics frameworks. By integrating GWAS with high-resolution transcriptomic data, our study advances beyond conventional single-method approaches. This integrative multi-omics strategy provides a more robust analytical framework to pinpoint candidate genes and elucidate the underlying molecular networks governing early-stage salt tolerance.

Although GWAS enables robust genomic localization, it often lacks the resolution to identify specific causal genes within large QTL intervals. Specifically, SNPs identified through GWAS often reside within broad linkage disequilibrium (LD) regions, making it challenging to distinguish whether a specific SNP is functionally causal or merely associated with the true causative locus. Furthermore, a single LD interval may encompass multiple genes, which often leads to potential false-positive results. Transcriptome analysis (RNA-seq) serves as a powerful complementary approach. Compared with traditional sequencing methods, second-generation transcriptomic sequencing provides higher sensitivity, greater throughput, and the ability to detect novel genes and alternative splicing events [28,29]. Rather than acting merely as a confirmatory tool, integrating transcriptional profiling with GWAS serves as a rigorous prioritization strategy. By determining the expression levels of genes within associated loci, this approach filters out non-responsive candidates within LD blocks and narrows the scope for biological validation, thereby significantly enhancing the accuracy of gene identification.

Consequently, integrating GWAS and transcriptomics has become an effective strategy for the genetic dissection of complex traits. Applying this combined approach to identify salt-tolerant genes and design molecular markers is crucial for enhancing cotton’s resilience. For instance, Xu et al. [30] conducted GWAS on 217 upland cotton varieties and, by incorporating transcriptomic data, identified three candidate genes associated with salt tolerance. Similarly, He et al. [31] combined these methods to identify 8 candidate genes associated with salt stress in alfalfa (*Medicago sativa* L.). Kim et al. [32] also utilized this integrated approach to predict key transcription factors (e.g., *OsMYB48*) associated with salt tolerance in rice seedlings. Beyond identification, high-confidence candidate genes derived from such multi-omics integration serve as optimal targets for CRISPR/Cas9-based functional validation. By developing knockout or overexpression models, the biological functions of these genes can be rapidly characterized. Furthermore, these functionally validated genes provide a theoretical basis for precision breeding, enabling the targeted enhancement of salt-resilience while preserving the elite genetic backgrounds of existing cultivars. This holistic pipeline, from locus discovery to potential application, significantly strengthens the translational value of genomic research in cotton.

This study utilized 300 landrace cotton germplasm accessions as experimental materials to investigate the genetic basis of salt tolerance. To address the previously identified knowledge gaps, our research was designed with the following specific objectives: (1) to characterize the phenotypic variation and stress response patterns in salt tolerance during the critical cotton germination stage; (2) to identify significant genomic loci and associated SNPs through a high-resolution GWAS; and (3) to prioritize high-confidence candidate genes by integrating genetic mapping findings with transcriptomic data. Collectively, these investigations aim to elucidate the molecular mechanisms underlying cotton salt tolerance and provide a robust theoretical foundation for the development of salt-resilient cotton cultivars through precision breeding.

## 2. Results

### 2.1. Phenotypic Analysis

**Figure A1:**
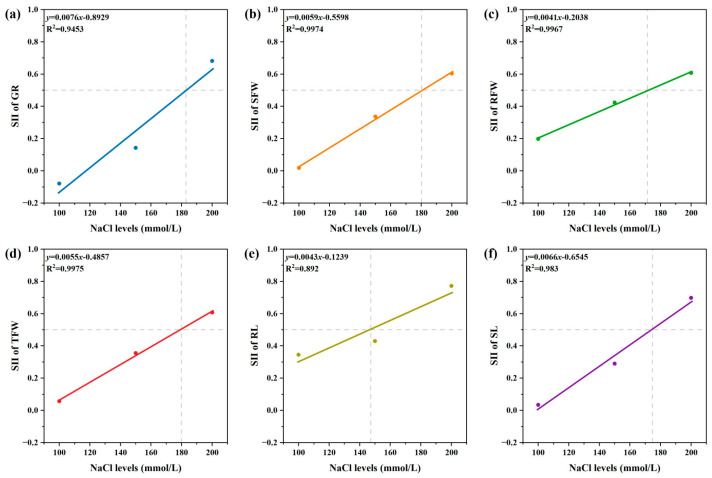
Selection of the optimal salt concentration for cotton germplasm salt tolerance evaluation. (**a**) Linear regression between SII of GR and NaCl concentrations. (**b**) Linear regression between SII of SFW and NaCl concentrations. (**c**) Linear regression between SII of RFW and NaCl concentrations. (**d**) Linear regression between SII of TFW and NaCl concentrations. (**e**) Linear regression between SII of RL and NaCl concentrations. (**f**) Linear regression between SII of SL and NaCl concentrations. The horizontal dashed line indicates SII = 0.5, and the vertical dashed line indicates the theoretical NaCl concentration when SII reaches 0.5.

A gradient stress test on 16 randomly selected cotton accessions revealed that the Salt Injury Index (SII) for six traits approached 0.5 under 200 mmol L^−1^ NaCl (Figure A1); thus, this concentration was selected for subsequent experiments. Salt Tolerance Indices (STIs) were calculated for the six traits and normalized using the membership function method. The average membership function value was used as the overall criterion for evaluating salt tolerance. Based on these values, the 300 germplasm accessions were classified into five categories: Highly Salt-Tolerant (HST, 48 accessions), Salt-Tolerant (ST, 169), Moderately Salt-Tolerant (MST, 40), Low Salt-Tolerant (LST, 16), and Non-Salt-Tolerant (NST, 27) [33]. This classification was subsequently utilized in downstream analyses to evaluate the enrichment and distribution of specific candidate gene haplotypes across accessions with differing salt tolerance capacities.

**Figure 1:**
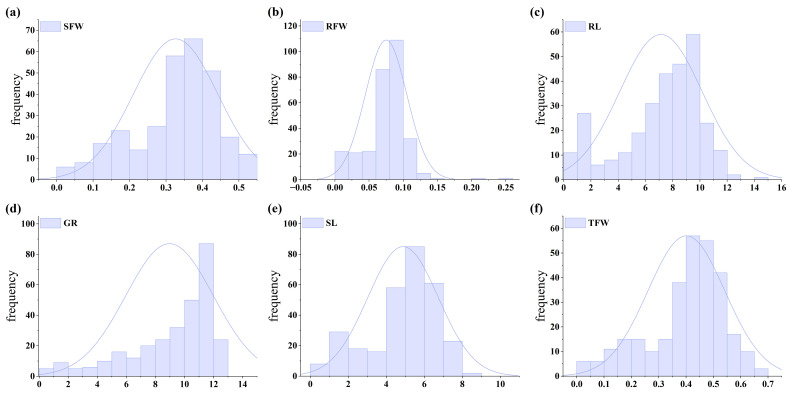
Phenotypic distribution of salt tolerance traits under NaCl stress. (**a**) Shoot Fresh Weight (SFW); (**b**) Root Fresh Weight (RFW); (**c**) Root Length (RL); (**d**) Germination Rate (GR); (**e**) Shoot Length (SL); (**f**) Total Fresh Weight (TFW). The curves represent the fitted normal distribution.

**Figure 2:**
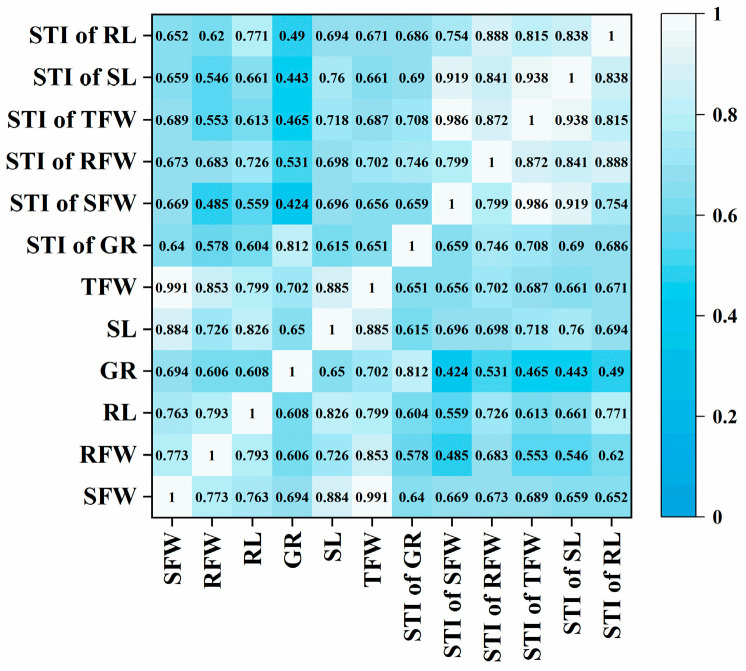
Correlation analysis among phenotypic traits and salt tolerance indices under NaCl stress.

Descriptive statistics (Table 1) showed that the coefficients of variation (CV) for these traits ranged from 31.93% to 48.52% under salt stress, indicating substantial phenotypic variation among the accessions. Normality tests (Figure 1) indicated that most traits followed a normal distribution (absolute skewness and kurtosis < 1), except for Germination Rate (GR) and Root Fresh Weight (RFW), which deviated from normality. These findings suggest that osmotic stress differentially regulates salt tolerance traits. While GR and RFW deviated from normal distributions, the General Linear Model (GLM) and Mixed Linear Model (MLM) employed in our GWAS are generally robust to such moderate non-normality, mitigating potential biases in the association results. Correlation analysis (Figure 2) revealed positive correlations among all traits. Total Fresh Weight (TFW) and Shoot Fresh Weight (SFW) exhibited the highest correlation coefficient (0.99), followed by the correlation between the STIs of TFW and SFW (0.99). The weakest correlation was observed between the STI of SFW and GR (r = 0.42).

### 2.2. Genome-Wide Association Analysis of Salt Tolerance-Related Traits in Cotton

Understanding population structure is critical for inferring the origin, differentiation, and gene flow among subpopulations. ADMIXTURE analysis was used to construct the population genetic structure. The cross-validation (CV) error was minimized at K = 6, yielding six subpopulations from the 300 cotton accessions. Specifically, the six subpopulations (Subpopulations 1 to 6) consisted of 57, 36, 28, 85, 69, and 25 accessions, respectively. Analysis of the geographical distribution indicated a correspondence between the population structure and the geographic origins of the accessions. Subpopulation 1 was primarily concentrated in the Northwest Inland region, Subpopulation 2 in the Yangtze River basin, and Subpopulation 6 in the Liaoning specific early-maturing region. The remaining subpopulations (3, 4, and 5) were predominantly located in the Yellow River basin. This classification was further corroborated by phylogenetic analysis and PCA. Based on PopLDdecay, the maximum r^2^ was 0.86, and the linkage disequilibrium (LD) decay distance (where r^2^ drops to half) was calculated as 454.6 kb [34]. This LD decay distance is shorter than the 742.7 kb reported by Ma et al. [35] and the 1000 kb reported by Fang et al. [36], but longer than the 296 kb reported by Wang et al. [37].

**Figure 3:**
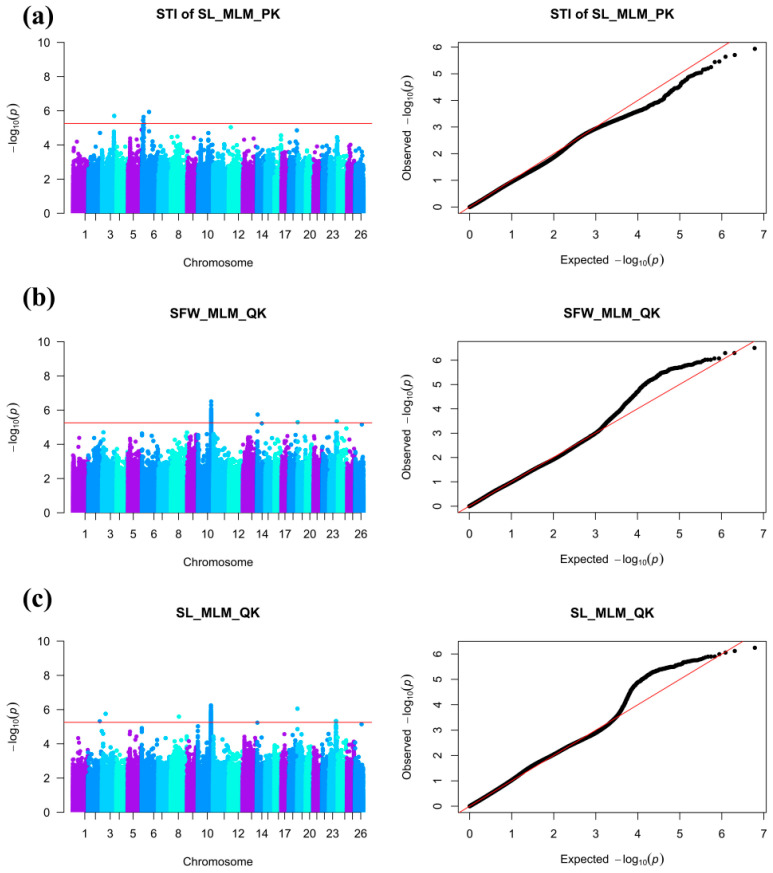
Manhattan plots and quantile-quantile (Q-Q) plots for three salt tolerance-related traits and indices. In the Manhattan plots, the x-axis represents the chromosomal position of SNPs, and the y-axis represents the significance of the association −log_10_(P). The red horizontal line indicates the significance threshold. In the Q-Q plots, the x-axis and y-axis represent the expected and observed −log_10_(P) values, respectively. (**a**) Salt Tolerance Index of Shoot Length (STI of SL); (**b**) Shoot Fresh Weight (SFW); (**c**) Shoot Length (SL).

GWAS was conducted for salt tolerance traits and indices using four models (GLM+P, GLM+Q, MLM+P+K, and MLM+Q+K) based on the identified effective SNPs (Figure 3). A total of 1277 significant SNPs were identified across 26 chromosomes, including 1111 for traits and 166 for indices. Based on the genomic LD decay distance of 454.6 kb, these SNPs were integrated into 43 QTLs for traits (explaining 6.71–11.88% of phenotypic variation, PVE, with an average of 8.03%) and 51 QTLs for indices (explaining 6.73–9.81% PVE, with an average of 7.38%). These moderate PVE values are consistent with the polygenic control of salt tolerance. Furthermore, they align with the typical effect range reported for salt tolerance QTLs in cotton by Yuan et al. [27] (5.98% to 10.76% PVE, with an average of 8.21%).

**Figure 4:**
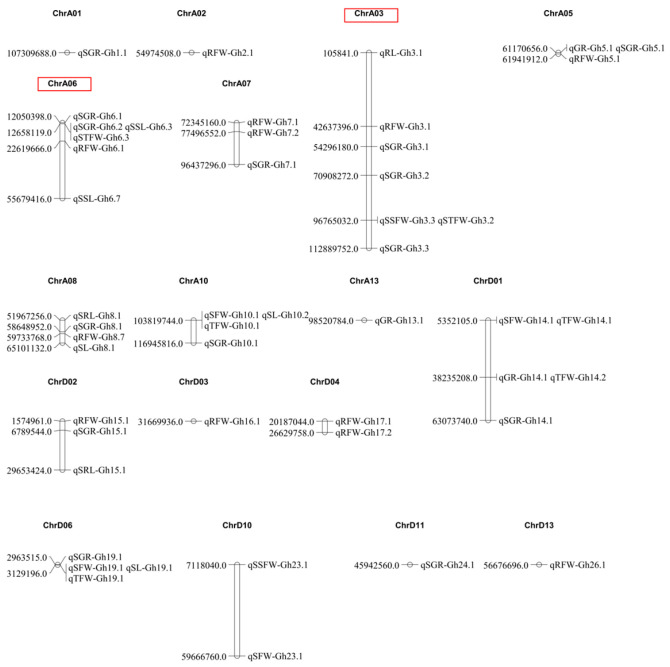
Chromosomal locations of QTLs associated with salt tolerance traits and salt tolerance indices. The red boxes highlight Chromosomes A03 and A06, which contain the highest number of stable QTLs.

To evaluate the robustness of these associations, we analyzed the detection frequency of the 94 identified QTLs across the four models. Specifically, 22 QTLs were uniquely detected by a single model, and 23 QTLs were shared across two models. Loci consistently detected by three or more models were explicitly defined as “stable QTLs”. Among the total, 49 stable QTLs were detected by at least three models (27 for traits and 22 for indices). As shown in Figure 4, these stable QTLs were distributed across most chromosomes, with the exception of A04, A09, A11, A12, D05, D07, D08, D09, and D12. Chromosome A03 contained the highest number of stable QTLs (7), followed by A06 (6), while A01, A02, A13, D03, D11, and D13 each contained one QTL. Specifically, stable QTLs associated with salt tolerance indices were most abundant on chromosome A03 (5), whereas those associated with salt tolerance traits were most abundant on chromosome D01 (4).

### 2.3. Candidate Gene Prediction

To identify candidate loci for salt tolerance, we prioritized QTLs from the 49 stable QTLs based on the following explicit selection criteria: (1) consistent detection across multiple GWAS models; (2) co-localization, where the same lead SNP is associated with multiple phenotypic traits; and (3) a high phenotypic variance explained (PVE). Applying these criteria, we focused on 9 prioritized QTLs where multiple traits were associated with the same lead SNP (Table 2). Specifically, *snp517602* was associated with *qSGR-Gh6.2*, *qSSL-Gh6.3*, and *qSTFW-Gh6.3*; *snp1470334* with *qSFW-Gh10.1*, *qSL-Gh10.2*, and *qTFW-Gh10.1*; and *snp2322113* with *qSFW-Gh19.1*, *qSL-Gh19.1*, and *qTFW-Gh19.1*. For subsequent candidate gene prediction, the QTL with the highest PVE at each of these three loci was selected as the representative trait, which were *qSSL-Gh6.3*, *qSFW-Gh10.1*, and *qSL-Gh19.1*, respectively.

**Figure 5:**
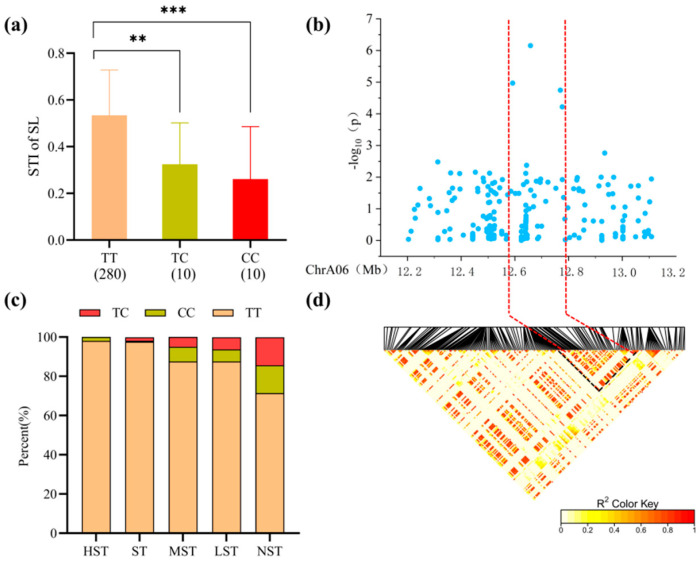
(**a**) Boxplot of Salt Tolerance Index (STI) for Shoot Length (SL) based on snp517602 genotypes; ** and *** indicate significant differences at the 0.01 and 0.001 levels, respectively. (**b**) Local Manhattan plot centered on the peak region of chromosome A06. (**c**) Stacked bar chart showing the percentage distribution of different haplotypes across salt tolerance categories. (**d**) Linkage disequilibrium (LD) heatmap of SNPs within the 12.2–13.1 Mb interval.

For *qSSL-Gh6.3* on chromosome A06 (Lead SNP: snp517602, PVE 7.94%), the local Manhattan plot (Figure 5b) peaked at 12.2–13.1 Mb. LD heatmap analysis (Figure 5d) narrowed the candidate region to 200 kb, containing three genes (Table A1). Haplotype analysis showed that accessions carrying the TT haplotype exhibited a significantly higher STI for Shoot Length compared to those with the CC or CT haplotypes. Moreover, frequency distribution analysis indicated that the TT haplotype accounted for the highest proportion in salt-tolerant categories, with the proportion decreasing as salt tolerance declined (Figure 5a,c).

**Figure 6:**
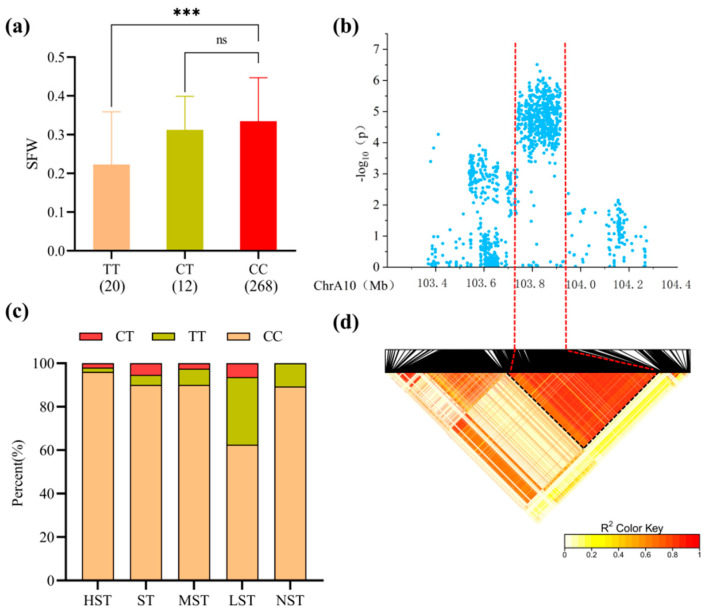
(**a**) Boxplot of Shoot Fresh Weight (SFW) based on snp1470334 genotypes; ns, not significant; *** indicates significant differences at the 0.001 level. (**b**) Local Manhattan plot centered on the peak region of chromosome A10. (**c**) Stacked bar chart showing the percentage distribution of different haplotypes across salt tolerance categories. (**d**) Linkage disequilibrium (LD) heatmap of SNPs within the 103.4–104.3 Mb interval.

For *qSFW-Gh10.1* on chromosome A10 (Lead SNP: snp1470334, PVE 8.43%), the peak was at 103.4–104.3 Mb. The 200 kb region contained 26 genes (Table A1). Accessions carrying the CC haplotype exhibited a significantly higher Shoot Fresh Weight, and distribution analysis showed this haplotype was most prevalent in salt-tolerant categories (Figure 6a,c).

**Figure 7:**
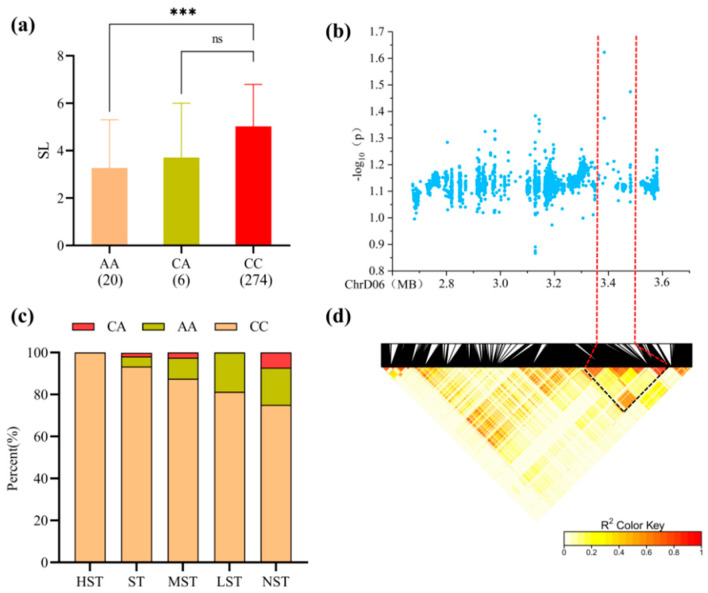
(**a**) Boxplot of Shoot Length (SL) based on snp2322113 genotypes; ns, not significant; *** indicates significant differences at the 0.001 level. (**b**) Local Manhattan plot centered on the peak region of chromosome D06. (**c**) Stacked bar chart showing the percentage distribution of different haplotypes across salt tolerance categories. (**d**) Linkage disequilibrium (LD) heatmap of SNPs within the 2.67–3.58 Mb interval.

For *qSL-Gh19.1* on chromosome D06 (Lead SNP: snp2322113, PVE 7.81%), the 150 kb region contained 44 genes (Table A1). Accessions carrying the CC haplotype exhibited significantly longer shoots, with the CC haplotype showing the highest proportion in salt-tolerant categories (Figure 7a,c).

### 2.4. Transcriptome Data Quality Analysis

It is important to note that the transcriptome analyses in this study were conducted exclusively on a single genotype and tissue. Five days post-germination, seedlings of the salt-tolerant accession CN4 (Zhongmian No. 4) were treated with 200 mmol L^−1^ NaCl or distilled water (control). Root tissues were collected at 1, 6, and 24 h with three biological replicates. Sequencing of the 18 libraries yielded 40.9–58.9 million clean reads per sample, with clean bases exceeding 6.13 Gb and an average GC content of 43.1%. The Q30 values for all samples were >96.49%, well above the standard threshold of 80%, demonstrating the high accuracy and reliability of the sequencing data. Furthermore, at least 95.87% of the clean reads were successfully mapped to the TM-1 reference genome (Table A2).

**Figure 8:**
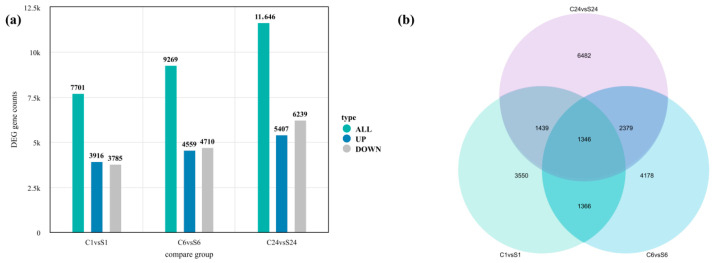
Statistical analysis of differentially expressed genes (DEGs) in roots under salt stress and control treatments. (**a**) Number of up-regulated and down-regulated DEGs at different time points. (**b**) Venn diagram showing the overlap of DEGs at different time points post-treatment.

At 1, 6, and 24 h post-salt stress treatment, a total of 7701 (3916 up-regulated and 3785 down-regulated), 9269 (4559 up-regulated and 4710 down-regulated), and 11,646 (5407 up-regulated and 6239 down-regulated) differentially expressed genes (DEGs) were identified between the stress and control groups, respectively (Figure 8a). Venn diagram analysis revealed overlaps between time points: 2712 DEGs were shared between 1 h and 6 h, 2785 between 1 h and 24 h, and 3725 between 6 h and 24 h. Notably, 1346 DEGs were common to all three time points (Figure 8b).

### 2.5. KEGG and GO Enrichment Analysis

**Figure 9:**
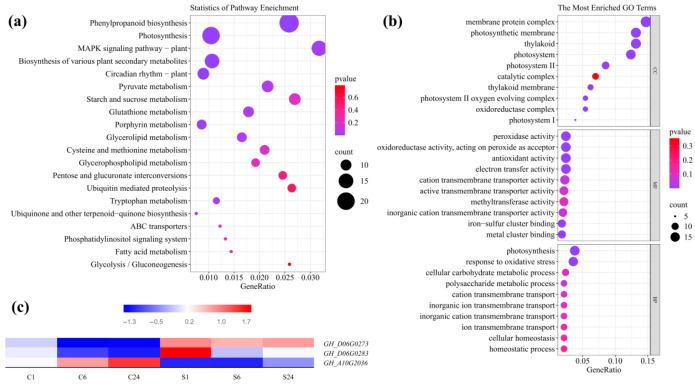
Enrichment and expression analysis of DEGs identified by RNA-seq. (**a**) KEGG pathway analysis of 1346 DEGs. (**b**) GO enrichment analysis of 1346 DEGs. (**c**) Expression levels of three DEGs at different sampling time points.

To monitor the expression of salt tolerance-related genes, KEGG and GO enrichment analyses were performed on the 1346 common DEGs. KEGG analysis indicated that these DEGs were enriched in 94 pathways. Figure 9a displays the top 20 significantly enriched pathways (*p* < 0.05). Most DEGs were involved in biosynthesis and metabolism, such as “Phenylpropanoid biosynthesis” (ghi00940, 24 DEGs), “Starch and sucrose metabolism” (ghi00500, 11 DEGs), “Pyruvate metabolism” (ghi00620, 11 DEGs), “Cysteine and methionine metabolism” (ghi00270, 9 DEGs), and “Biosynthesis of various plant secondary metabolites” (ghi00999, 15 DEGs). Additionally, several DEGs were associated with signal transduction, including “MAPK signaling pathway—plant” (ghi04016, 16 DEGs) and “Phosphatidylinositol signaling c” (ghi04070, 6 DEGs).

The Gene Ontology (GO) database classifies gene functions into Biological Process (BP), Cellular Component (CC), and Molecular Function (MF). We observed significant enrichment in 318 BP, 198 CC, and 78 MF terms (*p* < 0.05). Figure 9b lists the top 10 enriched terms in each category. For BP, the main enrichments were in “photosynthesis” (GO:0015979, 16 DEGs) and “response to oxidative stress” (GO:0006979, 15 DEGs). In CC, most DEGs were linked to “membrane protein complex” (GO:0098796, 19 DEGs) and “photosynthetic membrane” (GO:0034357, 17 DEGs). For MF, the highest numbers (16 DEGs each) appeared in “peroxidase activity” (GO:0004601), “antioxidant activity” (GO:0016209), “electron transfer activity” (GO:0009055), and “oxidoreductase activity, acting on peroxide as acceptor” (GO:0016684).

### 2.6. Screening Candidate Genes by Integrating GWAS and RNA-seq

To further screen for candidate genes associated with salt tolerance, we integrated the GWAS and RNA-seq results. Among the 73 candidate genes identified by GWAS, three overlapped with the common DEGs detected across all three sampling time points in the RNA-seq analysis (Table 3). Expression profiling revealed that these three candidates (GH_D06G0273, GH_D06G0283, and GH_A10G2036) exhibited significant differential expression (based on FPKM values) in all comparison groups (S1 vs. C1, S6 vs. C6, and S24 vs. C24) (Figure 9c). Consequently, these three genes were identified as prioritized candidate genes regulating salt tolerance during cotton germination within the specific context of the CN4 root transcriptome, precluding direct extrapolation to population-wide expression patterns.

## 3. Discussion

### 3.1. Optimization of Salt Stress Evaluation and Phenotypic Variation

Salt stress is a primary abiotic constraint limiting crop productivity, affecting over 1 billion hectares globally, with soil salinization in China showing an expanding trend [38]. Establishing an optimal stress intensity is the prerequisite for accurate germplasm evaluation. In this study, we determined that 200 mmol L^−1^ NaCl is the critical threshold for distinguishing salt tolerance in cotton germination. This selection is based on the principle that when the stress concentration ensures an intermediate phenotype (SII ≈ 0.5), the phenotypic variance within the population is often maximized. Such substantial variation is advantageous for GWAS as it facilitates the detection of genetic effects in polygenic traits while avoiding the excessive statistical bias caused by extreme concentrations (complete inhibition or lack of stress). This finding refines previous methodologies; for instance, Dilnur et al. [39] used 150 mmol L^−1^, while Sun et al. [38] found that 1.2% NaCl completely inhibited germination in sensitive accessions, ultimately selecting 1.0%. The variation in optimal concentrations across studies likely shoots from differences in the genetic background of the populations used. However, it must be acknowledged that the genetic networks and survival strategies activated at this semi-lethal dose may differ from plant responses under milder or field-relevant salinity conditions, where adaptive growth rather than immediate survival is the primary focus.

### 3.2. Identification of Stable QTLs Associated with Salt Tolerance

Seed germination is a critical bottleneck in the plant life cycle, yet most GWASs in cotton have focused on yield or fiber quality rather than early-stage abiotic stress tolerance [38,40]. Filling this gap, we identified 1277 significant SNPs and integrated them into 94 QTLs, with 49 designated as stable QTLs. The distribution of these QTLs was uneven, with a high density on chromosomes A03 and A06. While this clustering might suggest the presence of “resistance gene clusters,” such enrichment may also be partially influenced by the underlying genomic architecture, including local linkage disequilibrium (LD) decay patterns, marker density distribution, or the specific genetic composition of the natural population. Therefore, these regions should be viewed as high-priority genomic “hotspots” rather than definitive evidence of adaptive selection alone. Discrepancies in specific loci positions compared to other studies [41] suggest that salt tolerance is governed by distinct genetic networks that vary with germplasm diversity and the specific salt tolerance indices used. Future studies employing fine-mapping are required to further distinguish between these genomic structural effects and genuine biological signals.

### 3.3. Hormonal Regulation and Candidate Gene Functions

The integration of GWASs with transcriptomic data suggests a potential mechanistic understanding of how candidate genes regulate physiological responses. Our study highlights the central role of phytohormones, particularly Auxin and Abscisic Acid (ABA), in mediating salt stress adaptation.

On chromosome A06, the candidate gene *GH_A06G0675* was identified as a homolog of *YUC2* may be involved in auxin biosynthesis. Auxin is consistent with the alleviation of salt-induced oxidative damage by enhancing antioxidant enzyme activities [42,43,44]. On chromosome A10, *GH_A10G2042* encodes a RING-type E3 ligase homologous to Arabidopsis KEG, which is thought to negatively regulate ABA signaling by targeting ABI5 for degradation.

Therefore, these findings suggest a potential regulatory model for hormonal crosstalk during seed germination. Under salt stress, ABA accumulates as a core inhibitory signal, activating the downstream transcription factor ABI5 to maintain dormancy. Concurrently, YUC2-mediated auxin biosynthesis is initiated in active meristems such as the radicle, indirectly modulating ABA responses via ARF10/ARF16 [45]. KEG, acting as an E3 ubiquitin ligase, negatively regulates ABA signaling intensity by targeting ABI5 for degradation, thereby preventing excessive growth inhibition [46,47]. In this model, YUC2 and KEG synergistically regulate the balance of seed ABA responses through two dimensions: auxin synthesis flux and ABA signal attenuation rate. When environmental conditions become permissive, the reduction in ABA signaling and auxin-promoted radicle elongation jointly drive seed germination.

However, it is critical to state that these hormonal involvements are inferred solely from gene annotations and expression profiles. Because this study did not perform direct hormone measurements, signaling assays, or mutant analyses, these pathways should be regarded as proposed hypotheses rather than resolved mechanisms. Salt tolerance during germination likely involves complex multi-hormonal crosstalk that is currently beyond the resolution of this specific dataset.

### 3.4. Precision Mining of Key Regulators via Multi-Omics Integration

By using the intersection of GWAS candidates and RNA-seq DEGs as a prioritization filter, we identified three high-confidence candidate genes: *GH_D06G0273*, *GH_A10G2036*, and *GH_D06G0283*. This filtering approach relied on the consistency across independent genomic and transcriptomic datasets to prioritize targets for future functional validation, rather than providing direct evidence of causality. *GH_D06G0273* (encoding an LRR-RLK) is a prioritized candidate due to its role as a potential cell-surface sensor for stress signals [48]. Similarly, *GH_A10G2036* and *GH_D06G0283* represent high-confidence nodes in transcriptional reprogramming and protein turnover. Given that the 73 candidate genes were deterministically prioritized within highly specific local LD blocks (e.g., a restricted 200 kb interval for qSSL-Gh6.3) rather than randomly sampled, formal genome-wide enrichment probability models (such as the hypergeometric test) were not applicable. Instead, this transcriptomic integration served exclusively as a strict biological filter to resolve multi-gene co-segregation within these physically linked loci.

### 3.5. Study Limitations and Perspectives

This study has several limitations that delimit the scope of our conclusions. First, the RNA-seq analysis was performed using only a single salt-tolerant genotype (CN4) and focused exclusively on root tissue; thus, the identified expression patterns may not be universal across the entire population or across different tissues like leaves. Second, the identification of candidate genes relies heavily on Arabidopsis-based functional annotations, which may not perfectly overlap with cotton-specific gene functions. Third, while the effective SNP threshold used in GWAS strictly controls for false positives, it is highly conservative and likely overlooks numerous genuine but low-effect loci due to the assumptions of the Bonferroni correction in the presence of LD.

These candidate genes provide a foundation for future functional validation and targeted genome editing approaches (such as CRISPR/Cas9) aimed at improving salt tolerance in cotton. However, these loci should be treated as candidates for further research rather than ready-to-deploy targets for immediate breeding applications. Future work involving functional assays and multi-environment field trials is essential to confirm the stability and effect size of these candidates.

## 4. Materials and Methods

### 4.1. Materials

Three hundred cotton germplasm accessions were sourced from the China Germplasm Resource Bank, encompassing a wide range of ecological regions within China. The collection is widely distributed, comprising 99 accessions from the Yellow River cotton-growing region, 42 from the Yangtze River cotton-growing region, 89 from the inland northwest cotton-growing region, 43 from the ultra-early-maturing cotton-growing region of Liaoning, and 27 foreign germplasm accessions. These accessions cover a broad spectrum of genetic backgrounds and maturity types (early, mid, and late), providing diverse allelic variation for the association study.

### 4.2. Experimental Design

A subset of sixteen accessions was randomly selected from the 300-accession panel for a pilot study under four NaCl concentrations: 0, 100, 150, and 200 mmol L^−1^. The Salt Injury Index (SII) approached 0.5 at 200 mmol L^−1^ NaCl, which was subsequently chosen as the optimal concentration for large-scale evaluation. Seeds treated with sterile water (0 mmol L^−1^ NaCl) served as the control group. For each accession, thirty-six healthy seeds (twelve seeds per replicate, three replicates) with black seed coats were selected. Seeds underwent surface sterilization with 75% ethanol for one minute, followed by soaking in 4% sodium hypochlorite for eight minutes, and were rinsed five times with sterile water. The seeds were wrapped in two layers of filter paper saturated with the treatment solution and incubated in a growth chamber at 25 ± 1 °C and 65% relative humidity in darkness. Germination was recorded when the radicle length exceeded half the seed length. Seven days after germination, six traits were measured: germination rate (GR), root length (RL), shoot length (SL), root fresh weight (RFW), shoot fresh weight (SFW), and total fresh weight (TFW). In this study, the individual Petri dish served as the fundamental experimental unit to avoid pseudoreplication. For each biological replicate (i.e., one Petri dish), phenotypic measurements were obtained by averaging the results of the 12 seeds after excluding the maximum and minimum values to mitigate outliers. The mean value across the three biological replicates was subsequently calculated to represent the final phenotypic value for each accession, and these accession-level collective means were utilized as the direct input values for the downstream GWAS model. The salt tolerance index (STI) and salt injury index (SII) were calculated as described below:

(1) $$STI=\frac{Trait\;value\;under\;salt\;stress}{Trait\;value\;under\;control}$$

(2) SII=1−STI

(3) $$GR=\frac{Number\;of\;seeds\;germinated\;on\;day\;7}{Total\;number\;of\;seeds}\times 100\%$$

To ensure a thorough genetic dissection, GWAS was performed on both the raw traits under stress and their corresponding salt tolerance indices, as the latter accounts for inherent vigor differences across accessions under optimal conditions. Furthermore, a membership function method was employed to integrate these diverse traits into a single comprehensive score, allowing for a holistic assessment of salt tolerance.

The rationale for selecting 200 mmol L^−1^ NaCl for the main experiment was based on the need to maximize phenotypic variance within the diverse germplasm panel. A semi-lethal stress level, indicated by an SII of approximately 0.5, ensures that neither the resistant nor the susceptible accessions are severely limited in their physiological response, thus maintaining a wide range of phenotypic variation. This wide distribution of traits is critical for GWASs, as it increases the likelihood of detecting significant association signals related to salt tolerance mechanisms.

### 4.3. Data Processing

Data processing was conducted using Microsoft Excel 2016. Descriptive statistical analyses of phenotypic traits were carried out with SPSS Statistics 25. Frequency distribution histograms were generated in Origin 2022 to visually assess data distribution.

### 4.4. DNA Extraction and High-Throughput SNP Genotyping

Genomic DNA was extracted from fresh seedling leaves using the CWE9600 Magbead Blood DNA Kit (Cat. No. CWE9600, Kangwei Century, Beijing, China). Sequencing libraries were constructed through random fragmentation (300–350 bp), end-repair, PolyA tailing, and adapter ligation, followed by PCR amplification and purification. Libraries were sequenced on the DNBSEQ-T7 platform (BGI, Shenzhen, China) to generate 150 bp paired-end reads. Raw data were filtered using fastp [49] to remove adapters and low-quality reads (reads with >1% unknown bases or >50% bases with Q ≤ 5). Clean reads were aligned to the Gossypium hirsutum TM-1 reference genome (v2.1) using BWA-MEM (v0.7.17) [50]. Sorting and indexing were performed using Samtools (v1.7) [51]. PCR duplicates were marked using GATK MarkDuplicates. SNP calling was performed using the GATK (v4.1.8.0) HaplotypeCaller module [52]. The resulting VCF files were filtered using the VariantFiltration module with stringent criteria: individual missing rate ≤ 1%, SNP missing rate ≤ 1%, and minor allele frequency (MAF) > 0.05. Finally, 3,055,642 high-quality SNPs were retained for Principal Component Analysis (PCA), phylogenetic tree construction, population structure analysis, and GWASs.

### 4.5. Population Structure and LD Analysis

Genome-wide phylogenetic relationships were elucidated by constructing a Neighbor-Joining (NJ) tree using Tassel. Population genetic structure was assessed with Admixture software (v1.3.0), employing K values from 2 to 10 to investigate potential subdivisions within the panel, ranging from broader ecological groupings to finer sub-populations. The optimal number of clusters (K) was determined by identifying the value that minimized the cross-validation (CV) error. The results indicated that K = 6 provided the best fit for our data. Principal component analysis (PCA) was conducted using GCTA software (v1.94.1) to validate the ADMIXTURE results. Linkage disequilibrium (LD) decay was estimated by calculating the squared correlation coefficient (r^2^) between pairs of high-quality SNPs with PopLDdecay software (v3.43).

### 4.6. Genome-Wide Association Study

Genome-wide association studies (GWASs) were conducted using GEMMA (v0.94.1) [53] with four models: GLM (Q), GLM (P), MLM (Q+K), and MLM (P+K). The number of independent SNPs was determined using PLINK with the command --indep-pairwise 50 10 0.1, resulting in 182,147 effective SNPs [54]. The significance threshold was set at −log_10_(1/182,147) = 5.26. This Bonferroni-like threshold was utilized to maintain strict control over Type I error (false positives) amid the large volume of genetic markers tested. We acknowledge the inherent trade-off of this conservative approach, which may increase the risk of Type II error (false negatives) for minor-effect QTLs. To balance this, signals consistent across multiple models were also prioritized to complement the stringent statistical threshold. To minimize false-positive associations, loci detected by at least two models, particularly those consistent across both GLM and MLM frameworks, were prioritized for further analysis and candidate gene prediction. Manhattan and Q-Q plots were generated in R (v4.4.1) using the “ggplot2” and “qqman” packages, and LD heatmaps were produced with the “Ldheatmap” package.

### 4.7. Haplotype Analysis and Candidate Gene Prediction

After GWASs, significant SNPs for each trait were ordered by physical position. Adjacent SNPs were considered part of the same QTL if the physical distance between them was less than the estimated LD decay distance calculated in Section 4.5. For QTLs identified across multiple traits or markers, the SNP with the highest phenotypic variation explained (PVE) was selected for haplotype analysis. The Mann–Whitney U test was used to assess the significance of phenotypic differences between haplotypes. Candidate genes within the QTL interval defined by the LD decay distance were mapped to homologous genes in the Arabidopsis thaliana genome (www.arabidopsis.org, accessed on 20 April 2025) and further prioritized based on their functional annotation related to stress response and expression levels observed in our transcriptomic data.

### 4.8. Transcriptome Sequencing

Membership function clustering identified Zhongmian No. 4 (CN4), a high-salt-tolerant accession, for RNA sequencing. Seeds were germinated using the paper roll method for five days, then transferred to either 200 mmol L^−1^ NaCl or distilled water. Root tissues were collected at 1, 6, and 24 h post-treatment (labeled S1, S6, S24 for stress; C1, C6, C24 for control) and immediately frozen in liquid nitrogen. Three biological replicates were included for each treatment. While this design allowed for a detailed temporal analysis of the stress response in a representative tolerant genotype, we acknowledge it limits the generalizability of expression-based conclusions across the diverse cotton germplasm panel and other tissues. Library construction and sequencing were performed by Novogene Co., Ltd. (Beijing, China) on the Illumina HiSeq platform. Raw reads were filtered to remove adapters, N-containing reads, and low-quality reads (Qphred ≤ 5 bases accounting for >50% of read length). Clean reads were aligned to the *Gossypium hirsutum* TM-1 reference genome (v2.1) using HISAT2 [55] and assembled using StringTie [56] to reconstruct the transcriptome. Gene expression levels were estimated as Fragments Per Kilobase of transcript per Million mapped reads (FPKM). Data analysis was conducted using the Novogene NovoMagic platform. Differential expression analysis was performed with DESeq2 [57]. To control for the false discovery rate (FDR) in multiple testing, the *p*-values were adjusted using the Benjamini–Hochberg (BH) procedure. Differentially expressed genes (DEGs) were explicitly defined as those with an |Fold Change| ≥ 2 and FDR < 0.05. Specifically, DEGs within the previously identified QTL intervals were prioritized as potential candidate genes. Gene Ontology (GO) and Kyoto Encyclopedia of Genes and Genomes (KEGG) enrichment analyses were conducted using the Bioinformatics platform (http://www.bioinformatics.com.cn, accessed on 25 April 2025) to predict gene functions.

## 5. Conclusions

This study conducted a genome-wide association analysis using six salt tolerance traits and the salt tolerance index of 300 landraces of upland cotton under 200 mmol/L NaCl stress. A total of 1277 significant SNPs were detected and integrated into 94 QTLs, from which 49 stable QTLs were identified across multiple models. By utilizing transcriptomic data as a prioritization filter rather than proof of causality, we identified 73 genes potentially associated with salt tolerance, ultimately prioritizing three high-confidence candidate genes (*GH_D06G0273*, *GH_A10G2036*, and *GH_D06G0283*) for further investigation. It should be noted that these findings are hypothesis-generating and are currently limited by the use of a single genotype for transcriptome analysis and the absence of direct functional validation. Nonetheless, these prioritized candidates provide a valuable genomic resource and a foundation for future functional studies, including marker-assisted selection and precision genome-editing approaches (e.g., CRISPR/Cas) aimed at enhancing salt tolerance. Future research involving experimental validation and multi-environment trials will be essential to confirm these regulatory mechanisms, contributing to the development of climate-resilient cotton varieties.

## Figures and Tables

**Table 1:** Statistical description of phenotypic traits and salt tolerance indices under NaCl stress.

Trait	Mean	Min	Max	SD	CV (%)	Skewness	Kurtosis
SFW	0.326	0	0.54	0.116	35.61	−0.714	−0.059
RFW	0.075	0	0.26	0.031	40.72	0.203	4.72
RL	7.134	0	14.49	3.03	42.47	−0.784	−0.179
GR	0.748	0	1	0.252	33.7	−1.224	0.65
SL	4.88	0	8.71	1.858	38.08	−0.7	−0.231
TFW	0.401	0	0.69	0.141	35.19	−0.813	0.152
STI of GR	0.802	0	1.23	0.271	33.79	−1.327	0.928
STI of SFW	0.567	0	0.98	0.175	30.9	−0.529	0.495
STI of RFW	0.688	0	1.27	0.275	40.02	−0.837	0.063
STI of TFW	0.584	0	1.02	0.186	31.93	−0.64	0.387
STI of SL	0.519	0	0.99	0.203	39.16	−0.432	−0.327
STI of RL	0.708	0	1.45	0.344	48.52	−0.562	−0.592

Note: SFW, Shoot Fresh Weight; RFW, Root Fresh Weight; RL, Root Length; GR, Germination Rate; SL, Shoot Length; TFW, Total Fresh Weight; STI, Salt Tolerance Index; SD, Standard Deviation; CV, Coefficient of Variation.

**Table 2:** Stable QTLs detected across three replicates.

SNP	Position (bp)	QTL Name	Chr	−log_10_(P)	PVE (%)
snp517602	12,658,119	*qSGR-Gh6.2*	A06	5.58	7.15
		*qSSL-Gh6.3*	A06	6.15	7.94
		*qSTFW-Gh6.3*	A06	5.84	7.51
snp1470334	103,819,741	*qSFW-Gh10.1*	A10	6.51	8.43
		*qSL-Gh10.2*	A10	6.25	8.07
		*qTFW-Gh10.1*	A10	6.45	8.35
snp2322113	3,129,196	*qSFW-Gh19.1*	D06	5.42	6.93
		*qSL-Gh19.1*	D06	6.05	7.81
		*qTFW-Gh19.1*	D06	5.5	7.04

**Table 3:** Candidate genes for salt tolerance identified by integrating GWAS and RNA-seq.

Candidate Gene	Arabidopsis Homolog	Functional Description
*GH_D06G0273*	*AT1G09970*	Encodes RLK7, a leucine-rich repeat receptor-like kinase (LRR-RLK). It is involved in controlling germination speed and tolerance to oxidative stress. The mRNA is cell-to-cell mobile.
*GH_D06G0283*	*AT5G49665*	Encodes an E3 ubiquitin ligase involved in root gravitropism.
*GH_A10G2036*	*AT3G54390*	Encodes a sequence-specific DNA-binding transcription factor.

## Data Availability

Data are contained within the article.

## Abbreviations

The following abbreviations are used in this manuscript:

STI	Salt tolerance index
SII	Salt injury index
SL	Shoot length
RL	Root length
SFW	Shoot fresh weight
RFW	Root fresh weight
TFW	Total fresh weight (TFW)
GR	Germination rate

## Appendix A

**Table A1:** Candidate genes identified through GWAS for salt tolerance in cotton.

Chrom	Start	End	ID	Arabidopsis Homolog	Description
A06	12445389	12446496	GH_A06G0672	AT3G24520.1	member of Heat Stress Transcription Factor (Hsf) family
A06	12852645	12853394	GH_A06G0674	AT4G20520.1	RNA binding/RNA-directed DNA polymerase; (source:Araport11)
A06	12981260	12983438	GH_A06G0675	AT4G13260.1	Encodes YUC2. Catalyzes conversion of IPA (indole-3-pyruvic acid) to IAA (indole-3-acetic acid) in auxin biosynthesis pathway.
A10	103365525	103367502	GH_A10G2028	AT5G13500.3	Hyp O-arabinosyltransferase-like protein; (source:Araport11)
A10	103379058	103380601	GH_A10G2029	AT5G07030.1	Eukaryotic aspartyl protease family protein; (source:Araport11)
A10	103412773	103413123	GH_A10G2030	AT1G09620.1	ATP binding/leucine-tRNA ligases/aminoacyl-tRNA ligase; (source:Araport11)
A10	103414197	103415078	GH_A10G2031	AT1G67020.1	transmembrane protein; (source:Araport11)
A10	103417834	103418337	GH_A10G2032	AT5G19990.1	26S proteasome AAA-ATPase subunit The mRNA is cell-to-cell mobile.
A10	103426947	103430275	GH_A10G2033	AT3G61680.1	PLIP1 encodes a plastid localized phospholipase A1 involved in seed oil biosynthesis.
A10	103456127	103456840	GH_A10G2034	AT1G08315.1	ARM repeat superfamily protein; (source:Araport11)
A10	103748795	103750241	GH_A10G2035	AT2G43560.1	FKBP-like peptidyl-prolyl cis-trans isomerase family protein; (source:Araport11)
A10	103762102	103764641	GH_A10G2036	AT3G54390.1	sequence-specific DNA binding transcription factor; (source:Araport11)
A10	103793995	103797228	GH_A10G2038	AT3G06730.1	Encodes a plastidial thioredoxin (TRX) isoform that defines a branch of plastidial TRXs lying between x- and y-type TRXs and thus was named TRX z. Possesses disulfide reductase activity in vitro. TRX z was previously named as Trx p (thioredoxin putative and plastidic) before its thioredoxin activity was confirmed [58]. Knockout mutant of TRX z has a severe albino phenotype and is inhibited in chloroplast development. Two fructokinase-like proteins (FLN1 and FLN2), members of the pfkB-carbohydrate kinase family, are potential TRX z targets.
A10	103835582	103836670	GH_A10G2040	AT1G40087.1	Plant transposase (Ptta/En/Spm family); (source:Araport11)
A10	103876059	103881096	GH_A10G2041	AT3G12600.1	nudix hydrolase homolog 16; (source:Araport11)
A10	103901056	103913550	GH_A10G2042	AT5G13530.1	Encodes KEEP ON GOING (KEG), a RING E3 ligase involved in abscisic acid signaling. KEG is essential for Arabidopsis growth and development. ABA promotes KEG degradation via the ubiquitin dependent 26S proteasome pathway. Associates with and ubiquitinates MKK4 and MKK5 to regulate plant immunity.
A10	103960916	103963830	GH_A10G2043	AT4G02570.4	Encodes a cullin that is a component of SCF ubiquitin ligase complexes involved in mediating responses to auxin and jasmonic acid. Homozygous auxin-resistant mutants arrest growth soon after germination, lacking a root and hypocotyl. Heterozygotes display a variety of phenotypes consistent with impaired auxin response.
A10	103968662	103969084	GH_A10G2044	AT3G56710.2	Sig1 binding protein; interacts with Sig1R4. As well as Sig1, SibI is imported into chloroplasts and its expression is light-dependent in mature chloroplasts.
A10	104018056	104023180	GH_A10G2045	AT3G12250.7	basic leucine zipper transcription factor involved in the activation of SA-responsive genes.
A10	104024725	104031929	GH_A10G2046	AT1G20960.2	Similar to DEAD/DExH box ATP-dependent RNA helicase. Required for proper splicing of FLC. Mutants have reduced FLC levels and are early flowering.
A10	104035639	104036019	GH_A10G2047	AT1G76560.1	CP12 domain-containing protein 3; (source:Araport11)
A10	104040684	104041578	GH_A10G2048	AT4G08850.1	MIK1 encodes a receptor kinase that forms a complex with MDIS1/MIK2 and binds LURE1, the female pollen guidance chemi-attractant. MIK1 phosphorylates MDIS1 and is autophosphorylated. Also functions as a receptor for the phytocytokine SCOOP10 in regulation of leaf sensescense.
A10	104104672	104107793	GH_A10G2049	AT4G08850.1	MIK1 encodes a receptor kinase that forms a complex with MDIS1/MIK2 and binds LURE1, the female pollen guidance chemi-attractant. MIK1 phosphorylates MDIS1 and is autophosphorylated. Also functions as a receptor for the phytocytokine SCOOP10 in regulation of leaf sensescense.
A10	104114890	104115423	GH_A10G2050	AT2G13980.1	Polynucleotidyl transferase, ribonuclease H-like superfamily protein; (source:Araport11)
A10	104185042	104186989	GH_A10G2051	AT2G38730.1	Cyclophilin-like peptidyl-prolyl cis-trans isomerase family protein; (source:Araport11)
A10	104195294	104197311	GH_A10G2052	AT3G12260.1	LYR family of Fe/S cluster biogenesis protein; (source:Araport11)
A10	104198228	104200175	GH_A10G2053	AT2G38730.1	Cyclophilin-like peptidyl-prolyl cis-trans isomerase family protein; (source:Araport11)
A10	104214729	104214941	GH_A10G2054	AT5G11020.3	Protein kinase superfamily protein; (source:Araport11)
A10	104255076	104258258	GH_A10G2055	AT1G21230.1	encodes a wall-associated kinase The mRNA is cell-to-cell mobile.
D06	2700492	2730652	GH_D06G0266	AT4G39756.1	Galactose oxidase/kelch repeat superfamily protein; (source:Araport11)
D06	2883178	2886700	GH_D06G0267	AT1G59520.1	Encodes CW7.
D06	2889320	2891953	GH_D06G0268	AT1G59540.1	Encodes a kinesin-like protein.
D06	2892296	2893273	GH_D06G0269	AT5G03020.1	Galactose oxidase/kelch repeat superfamily protein; (source:Araport11)
D06	2898698	2899550	GH_D06G0270	AT5G41150.4	Confers resistance to UV radiation. Homolog of the human xeroderma pigmentosum group F DNA repair and yeast Rad1 proteins
D06	2926014	2958858	GH_D06G0271	AT1G58350.4	Putative serine esterase family protein; (source:Araport11)
D06	2969501	2972439	GH_D06G0272	AT5G49660.1	The gene encodes receptorlike kinase (RLK). Involved in the maintenance organization of cell files or cell morphology in conductive elements. Functions as a receptor for CEP1 peptide. Mediates nitrate uptake signaling.
D06	2976635	2979669	GH_D06G0273	AT1G09970.1	RLK7 belongs to a leucine-rich repeat class of receptor-likekinase (LRR-RLKs). It is involved in the control of germination speed and the tolerance to oxidant stress. The mRNA is cell-to-cell mobile.
D06	2996476	3001165	GH_D06G0274	AT1G09960.1	low affinity (10 mM) sucrose transporter in sieve elements (phloem)
D06	3006084	3007790	GH_D06G0275	AT1G58340.1	Encodes a plant MATE (multidrug and toxic compound extrusion) transporter that is localized to the Golgi complex and small organelles and is involved in determining the rate of organ initiation. It is also involved in iron homeostasis when plants are under osmotic stress.
D06	3018416	3018732	GH_D06G0276	AT3G51250.1	Senescence/dehydration-associated protein-like protein; (source:Araport11)
D06	3044464	3045471	GH_D06G0277	AT5G25200.1	Ta11-like non-LTR retrotransposon; (source:Araport11)
D06	3048805	3050713	GH_D06G0278	AT1G27600.2	Encodes a member of the GT43 family glycosyltransferases involved in glucuronoxylan biosynthesis: AT2G37090 (IRX9) and AT1G27600 (IRX9-L or I9H, IRX9 homolog); AT4G36890 (IRX14) and AT5G67230 (IRX14-L or I14H, IRX14 homolog). They form two functionally non-redundant groups essential for the normal elongation of glucuronoxylan backbone. I9H functions redundantly with IRX9, I14H is redundant with IRX14. IRX9 or I9H do not complement IRX14, IRX14 or I14H do not complement IRX9.
D06	3051821	3055359	GH_D06G0279	AT3G14470.2	NB-ARC domain-containing disease resistance protein; (source:Araport11)
D06	3056714	3060184	GH_D06G0280	AT3G14470.2	NB-ARC domain-containing disease resistance protein; (source:Araport11)
D06	3065933	3066910	GH_D06G0281	AT5G39560.1	Galactose oxidase/kelch repeat superfamily protein; (source:Araport11)
D06	3069947	3071763	GH_D06G0282	AT1G27530.1	ubiquitin-fold modifier-conjugating enzyme; (source:Araport11)
D06	3088914	3090986	GH_D06G0283	AT5G49665.1	E3 ubiquitin ligase involved in root gravitropism.
D06	3139671	3142157	GH_D06G0284	AT5G65670.2	auxin (indole-3-acetic acid) induced gene The mRNA is cell-to-cell mobile.
D06	3157430	3157906	GH_D06G0285	AT5G65660.1	hydroxyproline-rich glycoprotein family protein; (source:Araport11)
D06	3167323	3169215	GH_D06G0286	AT5G65640.1	bHLH093/NFL encodes a bHLH transcription factor involved in GA mediated control of flowering time. Mutants are non-flowering in short days and phenotype can be reversed with GA application. Based on the expression of GA biosynthetic genes in the mutant, it likely acts through regulation of GA metabolism. Its expression shows developmental stage and tissue specificity. In short days it is expressed mainly in root tips and SAM, with weak expression in cotyledons throughout development. In LD GUS activity was observed in the hypocotyl and in root tips and SAM throughout the developmental stages.
D06	3206884	3209746	GH_D06G0287	AT5G64460.8	Phosphoglycerate mutase family protein; (source:Araport11)
D06	3259887	3261427	GH_D06G0288	AT1G09935.1	Phosphoglycerate mutase family protein; (source:Araport11)
D06	3277421	3279502	GH_D06G0289	AT1G09935.2	Phosphoglycerate mutase family protein; (source:Araport11)
D06	3285398	3288960	GH_D06G0290	AT4G24400.1	Encodes a CBL (calcineurin B-like calcium sensor proteins) -interacting serine/threonine protein kinase. Regulates the low-affinity phase of the primary nitrate response. The mRNA is cell-to-cell mobile.
D06	3289497	3291903	GH_D06G0291	AT2G22070.1	pentatricopeptide (PPR) repeat-containing protein; (source:Araport11)
D06	3293828	3294124	GH_D06G0292	AT5G14895.1	NHL domain protein; (source:Araport11)
D06	3298439	3300226	GH_D06G0293	AT1G09900.1	Pentatricopeptide repeat (PPR-like) superfamily protein; (source:Araport11)
D06	3316248	3331591	GH_D06G0294	AT3G57230.4	MADS-box transcription factor. Expressed in leaf, root and stem, with higher RNA accumulation in guard cells and trichomes. AGL16 can directly interact with SVP and indirectly interact with FLC. Furthermore, the accumulation of AGL16 transcripts is modulated by miR824 (AT4G24415). The flowering time effect for the miR824/AGL16 module is more obvious in the Col-FRI background than in the Col-0 background. AGL16 controls flowering via an allelic dosage effect in long-day non-vernalized conditions.AGL16 binds to promoters of over 2000 genes with CArG-box motifs. Forms a complex with SOC1 and regulates its expression.
D06	3351581	3353768	GH_D06G0295	AT1G13950.1	Encodes eukaryotic translation initiation factor 5A (EIF-5A).In mammalian cells it functions as a shuttle protein that translocates mRNA from the nucleus to cytoplasmic ribosomes. Overexpression results in an increase in both primary and secondary xylem formation. In RNAi suppressed lines, xylem formation is reduced.
D06	3356295	3359350	GH_D06G0296	AT1G09920.1	TRAF-type zinc finger-like protein; (source:Araport11)
D06	3368501	3383565	GH_D06G0297	AT1G09890.4	Rhamnogalacturonate lyase family protein; (source:Araport11)
D06	3387995	3402547	GH_D06G0298	AT1G55860.1	encodes a ubiquitin-protein ligase containing a HECT domain. There are six other HECT-domain UPLs in Arabidopsis.
D06	3404137	3406609	GH_D06G0299	AT1G74520.2	Part of the AtHVA22a family. Protein expression is ABA- and stress-inducible.
D06	3408592	3410230	GH_D06G0300	AT5G09570.1	Twin CX9C domain protein. Induced by low phosphate or iron, drought and heat stress. Loss of both At12cys-1 and At12cys-2 lead to enhanced tolerance to drought and light stress and increased anti-oxidant capacity.
D06	3415615	3419580	GH_D06G0301	AT3G21175.2	member of a novel family of plant-specific GATA-type transcription factors.
D06	3432374	3436121	GH_D06G0302	AT4G24470.3	ZIM is a putative transcription factor containing an atypical GATA-type zinc-finger motif.
D06	3440152	3441564	GH_D06G0303	AT5G49690.1	UDP-glycosyltransferase that can act upon sulcotrione herbicide. Overexpression confers resistance to herbicide.
D06	3515091	3521896	GH_D06G0304	AT4G24480.2	Raf-like kinase required for stomatal vapor pressure difference response.
D06	3525314	3525780	GH_D06G0305	AT1G58225.1	hypothetical protein; (source:Araport11)
D06	3528995	3532669	GH_D06G0306	AT4G24490.2	RAB geranylgeranyl transferase alpha subunit 1; (source:Araport11)
D06	3545640	3546926	GH_D06G0307	AT5G67150.1	HXXXD-type acyl-transferase family protein; (source:Araport11)
D06	3566453	3568229	GH_D06G0308	AT1G09700.2	Encodes a nuclear dsRNA binding protein. Involved in mRNA cleavage. The mutant is characterized by shorter stature, delayed flowering, leaf hyponasty, reduced fertility, decreased rate of root growth, and an altered root gravitropic response. It also exhibits less sensitivity to auxin and cytokinin.
D06	3583673	3587126	GH_D06G0309	AT1G58215.1	spindle pole body component 110; (source:Araport11)

**Table A2:** Summary of sequencing data quality.

SNP	Position (bp)	QTL Name	Chr	−log_10_(P)	PVE (%)
C1_1	40,886,550	6,132,982,500	43.19	97.09	97.39
C1_2	44,402,422	6,660,363,300	43.02	96.91	95.98
C1_3	47,906,788	7,186,018,200	42.95	97.24	95.87
C6_1	45,309,922	6,796,488,300	42.99	97.09	96.66
C6_2	42,474,010	6,371,101,500	43.03	97.11	96.61
C6_3	41,495,288	6,224,293,200	42.93	97.09	96.38
C24_1	45,281,636	6,792,245,400	42.87	97.21	96.85
C24_2	58,868,188	8,830,228,200	42.8	97.09	96.29
C24_3	43,792,886	6,568,932,900	42.86	97.06	96.85
S1_1	43,809,246	6,571,386,900	42.28	96.49	95.87
S1_2	44,405,540	6,660,831,000	42.79	97.12	98.11
S1_3	41,920,684	6,288,102,600	43.06	96.94	97.57
S6_1	41,956,928	6,293,539,200	43.89	97.04	97.54
S6_2	44,813,784	6,722,067,600	43.15	96.94	98
S6_3	43,704,750	6,555,712,500	43.1	96.95	97.8
S24_1	44,887,390	6,733,108,500	43.36	96.93	97.64
S24_2	44,219,242	6,632,886,300	43.45	97.18	97.66
S24_3	44,245,894	6,636,884,100	43.23	97.03	97.79
